# On and Off Deformability of Supramolecular Micelles in the Soft Frank–Kasper σ Phase

**DOI:** 10.1021/acs.jpclett.6c00287

**Published:** 2026-03-20

**Authors:** Shih-Yong Chen, Chin-Hong Goh, Mayumi Egashira, Chun-Yu Chen, Jhih-Min Lin, Chien-Lung Wang

**Affiliations:** † Department of Chemistry, 33561 National Taiwan University, No. 1, Sec. 4, Roosevelt Road, Taipei 10617, Taiwan; ‡ 57815 National Synchrotron Radiation Research Center, 101 Hsin-Ann Road, Hsinchu 30076, Taiwan

## Abstract

Deformability is a key pathway to structural complexity in self-assembled systems. Although numerous molecular systems have been engineered to form Frank–Kasper (FK) phases, they typically rely on size- or shape-based asymmetries rather than new structural degrees of freedom. By incorporating chain-length asymmetry into dendritic amphiphiles, this study endows supramolecular micelles in the soft FK σ phase with a controllable deformability. The asymmetric dendrons (ADs) form anisotropic σ phases with domain-dependent diffraction patterns caused by micelle deformation, as verified by the anisotropic Debye–Waller simulations. Over time, these anisotropic micelles reorganize along specific planes, triggering a σ-to-lamellar transition. Incorporating hydrophobic guest molecules compensates for the chain-length asymmetry, switching off deformability and restoring isotropic packing. Thus, deformability emerges as a tunable parameter governing lattice symmetry and phase evolution, offering a design principle for scalable and hierarchically complex assemblies.

## Graphical abstract

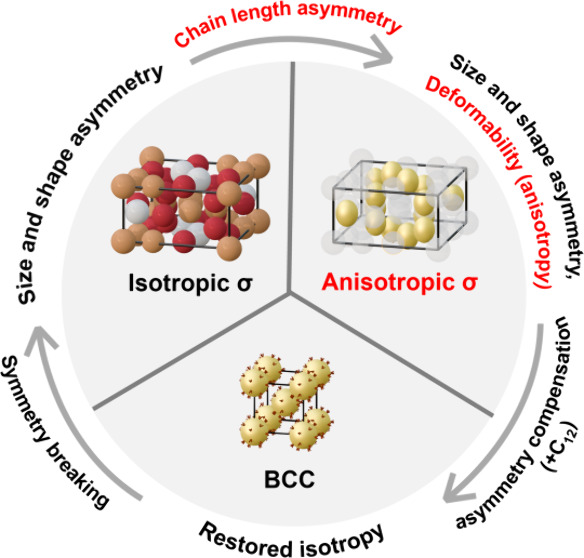

Nature creates self-assembling systems that are not only precise and dynamic but also capable of evolving structural complexity essential for biological functions.^1^ In these systems, complexity does not simply arise from molecular diversity, but from cooperative interactions and adaptive architectures operating across multiple hierarchical levels.^2,3^ As structural modules deform and reorganize in response to spatial constraints or interaction cues, they give rise to complex architectures and emergent functionalities that are absent at the individual molecular level. The deformability of biological macromolecules thus represents a key principle through which nature achieves higher-order organization.^4,5^ By enabling continuous modulation of local geometries, proteins can reorganize into distinct supramolecular architectures that perform specific collective functions.^6^

To reach such complex supramolecular structures, various asymmetries have been applied to synthetic self-assembly systems to shape their morphology.^7–9^ For example, the uneven volume ratios of the incompatible building blocks lead to compositional asymmetry in block copolymers, enabling the formation of 0D spheres, 1D cylinders, 2D lamellae, and 3D interpenetrating supramolecular structures.^10,11^ In addition, differences in the number and physical properties of the incompatible blocks cause conformational asymmetry at the molecular level,^12–14^ which in turn induces packing frustration and generates micelles exhibiting size and shape asymmetry in the bulk state.^15,16^ This hierarchical asymmetry results in structural complexity at the supramolecular level and ultimately drives the formation of Frank–Kasper (FK) phases, a class of complex spherical structures.^17–19^ Moreover, analogous compositional and conformational asymmetries have been leveraged in the design of dendritic^20–24^ and giant molecules.^25–28^ Their structural tunability facilitates the construction of intricate molecular architectures,^29–31^ allowing for precise control over the formation of complex supramolecular structures.^32,33^

Although numerous molecular systems have been designed to form FK phases, it seems that the variety of molecular architectures has not always been hierarchically transformed to exhibit variable complexity at the supramolecular level. With the FK σ phase as an example, similar small-angle X-ray scattering (SAXS) patterns were obtained from different molecular systems,^25,34–38^ suggesting that the supramolecular micelles of the very distinct synthetic building blocks reached similar degrees of size and shape asymmetry in the σ phases. This observation raises a fundamental scientific question, echoing Lehn’ s principle regarding which molecular designs are essential to progressively achieve structural complexity.^3^ To realize such complexity in practice, however, it is equally important to identify mechanisms that stabilize FK structures and prevent reversion to simpler cubic packing states.

In soft matter systems, the stabilization of the FK phases involves breaking the symmetry of spherical micelles. The result of symmetry breaking in the literature creates micellar polyhedra with a size and shape distribution,^39,40^ which does not significantly change the isotropic shape of the polyhedra. Theoretical studies have revealed that elongating spherical micelles into ellipsoidal shapes can also effectively stabilize FK phases.^41^ Such geometric anisotropy reduces the extent of packing frustration and offers an alternative pathway to FK phase stabilization. A unique soft FK phase was constructed with mesoatoms, where a degree of shape anisotropy was also recently observed by Cheng and co-workers,^42^ showing that equipping the FK phase with structural anisotropy may create possibilities of reaching structural complexity beyond that of current FK phases. However, the mechanism of elongation stabilization has yet to be fully clarified due to the current lack of more sophisticated structural characterization tools and a deeper understanding of the building blocks.

Inspired by the stabilization effect of micelle elongation, in this study, we applied chain-length asymmetry to a dendritic molecule and examined if the supramolecular micelles built with the asymmetric dendron (AD) in Scheme 1 can be deformed into anisotropic geometries without degrading the complexity in the FK phase. Similar to the symmetric dendron (SD) in our previous study,^43^ an AD is an amphiphilic, cone-like molecule with equal molecular weight. Upon self-assembly, symmetry breaking forced the SD micelles into the typical FK σ phase. We hypothesized that the chain-length asymmetry of AD would generate softer micelles with enhanced deformability, thereby enabling the formation of anisotropic supramolecular micelles within the FK phase. This property may allow the AD to introduce anisotropy into the FK phase through elongation stabilization.

**Scheme 1:**
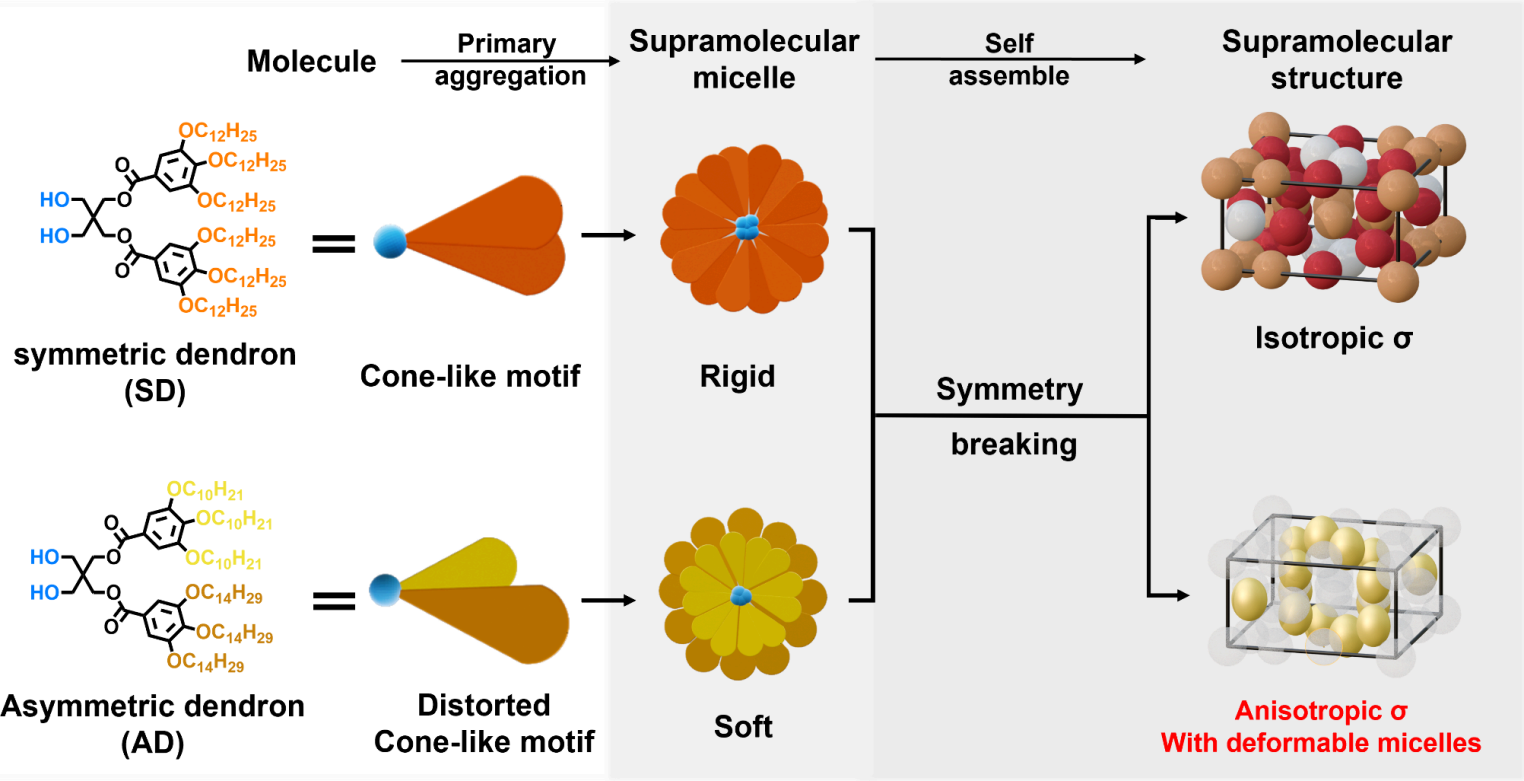
Illustration of the Influence of Chain-Length Asymmetry on the Complexity of Supramolecular Self-Assembly^a^

a The symmetric dendron (SD) forms compact supramolecular micelles with limited deformability, leading to the formation of an isotropic FK σ phase. In contrast, the asymmetric dendron (AD) forms softer micelles with enhanced deformability, giving rise to a novel anisotropic FK σ phase.

Scheme S1 shows the synthetic route of the AD. In contrast to the previously reported SD, the AD was designed with chain-length asymmetry by incorporating 3,4,5-tris­(decyloxy)­benzoyl and 3,4,5-tris­(tetradecyloxy)­benzoyl dendrons. The ^1^H NMR, ^13^C NMR, and mass spectra shown in Figures S1–S6 confirm the molecular structure of the AD. Figure S7 shows the in situ temperature-dependent small-angle X-ray scattering (SAXS) data that reveal the phase behavior of AD. In the first heating scan, the AD carried out the phase transition from the L phase to the dodecagonal quasicrystalline phase (DDQC), then the FK σ phase, and finally the melt state. Subsequent cooling moved the AD from the melt back to the σ phase, and in the second heating, only the σ-to-melt phase transition was observed. Similar to that of the SD,^43^ the σ phase of the AD is actually a kinetically accessible metastable phase, as aging the σ phase under the ambient condition for 3 weeks eventually moved the AD from the σ phase to the L phase, as shown in Figure S8. The isotropization temperature (*T*_i_) of the AD identified from the in situ SAXS results is 56 °C, about 10 °C lower than that of the SD. This indicates that although the AD exhibits phase behavior similar to that of the SD, the chain-length asymmetry of AD destabilizes the σ phase. Despite the decreased *T*_i_, as shown in Figure S9 and Table S1, the AD still forms a σ phase with lattice parameters close to those of the SD.

However, within the nearly identical tetragonal lattices, the constituent supramolecular micelles of the AD are completely different from those of the SD. The supramolecular micelles of the SD represent the typical micelles that only slightly deviate from a perfect sphere in geometry. Due to the isotropy of the micelles in the FK lattice (Figure 1a), the three representative microbeam synchrotron 2D-SAXS patterns (incident beam size of 5 μm × 5 μm) collected from different grains of the SD’s σ phase show nearly identical intensity distributions for the [*hkl*] diffractions. To make the comparison easy, the intensities of the diffraction signals in the microbeam SAXS patterns were integrated and are shown as the 1D SAXS patterns in Figure 1b. The identical intensity profile indicates that the SD micelles in the σ phase lack geometrical anisotropy to alter the intensity distribution of the [*hkl*] peaks.^44^ In contrast, in panels c and d of Figure 1, the three representative 2D microbeam SAXS patterns and six integrated 1D SAXS patterns out of 10 measurements of the σ phase of the AD give completely different diffraction intensity profiles from distinct crystalline grains of AD, even though all of the SAXS patterns were collected from the identical [11̅0] zone axis of the σ phase according to the zonal equation (detailed crystallographic information and zone axis analysis are provided in Table S2 and eq S3, respectively). Since the Laue condition defines the *q*_*hkl*_ of constructive X-ray interference, and the geometry and orientation of the motifs within the lattice determine the intensity profiles of the X-ray diffraction from a lattice,^44^ for the completely different intensity ratios of the AD in the σ phase to be nearly identical to that of the SD, the geometry of the AD micelles must significantly deviate from an isotropic shape. Theoretical study has indicated that in addition to the size and shape asymmetry, the FK phases can also be stabilized by the anisotropy of the constituent motifs.^41^ As the intensity profile of diffraction is sensitive to the changes in the orientation of anisotropic motifs in a lattice, it is possible that the chain-length asymmetry of the AD enables the formation of supramolecular micelles with a high degree of deformability. This deformability of the micelles leads to symmetry breaking beyond the well-known size and shape asymmetry, resulting in anisotropic characteristics in the σ phases formed by AD molecules.

**Figure 1:**
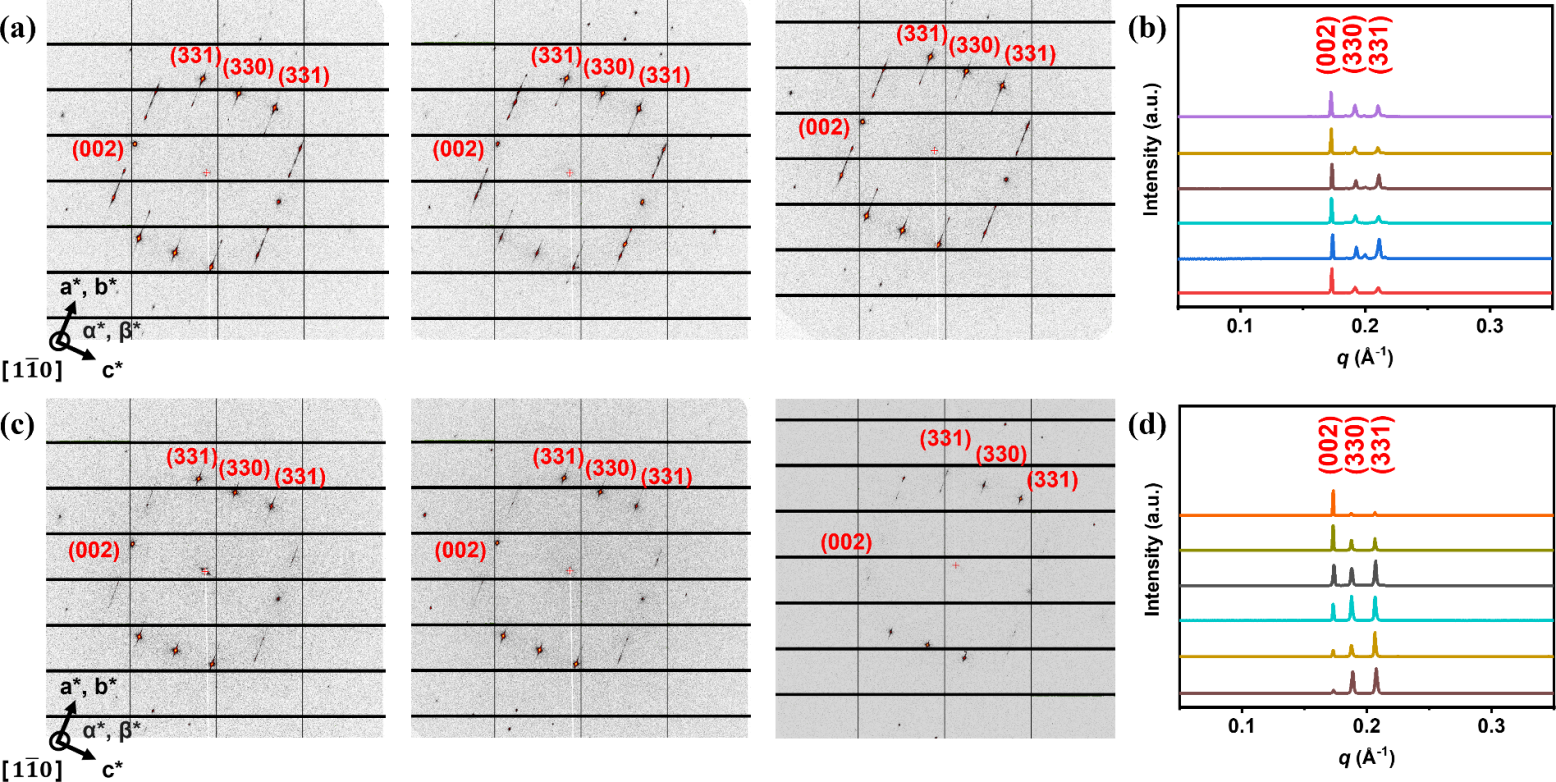
(a) Microbeam SAXS patterns collected from different domains of the σ phase of the SD from the [11̅0] zone. (b) Integrated intensity profiles of the SAXS patterns shown in panel a. (c) Microbeam SAXS patterns collected from different domains of the σ phase of the AD from the [11̅0] zone. (d) Integrated intensity profiles of the SAXS patterns shown in panel c.

1 $$F\left(hkl\right)=\underset{j}{\sum }f_{j}\times e^{\left(2\pi i\left(hx_{j}+ky_{j}+lz_{j}\right)\right)}\times e^{\left(-\left(8\pi^{2}U_{i}{\;\sin}^{2}\;\theta \right)/\lambda^{2}\right)}$$

2 $$I\left(hkl\right)\propto {\left|F\left(hkl\right)\right|}^{2}$$

3 $$F_{\sigma }\left(hkl\right)=\overset{30}{\underset{j=1}{\sum }}f_{j}^{\left(\mathrm{motif}\right)}\times e^{\left(2\pi i\left(hx_{j}+ky_{j}+lz_{j}\right)\right)}\times e^{\left(-\left(8\pi^{2}U_{j}^{\left(\mathrm{motif}\right)}{\;\sin}^{2}\;\theta \right)/\lambda^{2}\right)}$$

The diffraction characteristics of the σ phase lattice can be rationalized using the classical structure factor formalism (eqs 1 and 2),^44^ where the scattering intensity is governed by intrinsic scattering factor *f*_*j*_, the phase factor from spatial coordinates (*x*_*j*_, *y*_*j*_, *z*_*j*_), and the Debye–Waller term representing thermally induced displacements. In the σ phase formed by the AD, however, the supramolecular motifs are fluidic aggregates composed of multiple AD molecules (≈19 ADs per micelle). The positions of the constituent atoms within the tetragonal framework are thus not fixed; accordingly, the “coordinates” in the phase term are more appropriately interpreted as the centers of mass of the supramolecular micelles.^44^ In this context, micelles derived from chain-length asymmetric AD molecules occupy the σ phase lattice sites and act as supramolecular scattering motifs rather than atomic scatterers. By replacing atomic scattering factor *f*_*j*_ with a motif-level scattering factor *f*_*j*_^(motif)^ and mapping the 30 crystallographic sites of the σ phase into the phase factor,^45^ the structure factor can be reformulated as eq 3, where the Debye–Waller term serves as a tunable descriptor encoding the deformability of the fluidic motifs within the lattice.

To examine whether this deformation term can rationalize the atypical reflection:intensity ratios observed experimentally, we constructed an isotropic σ phase model representing SD micelles and two anisotropic σ phase models featuring deformation along the *c*-axis or within the *a–b* plane (Figure 2a). Single-zone diffraction simulations were carried out in Cerius^2^, using the temperature factor as the sole adjustable parameter (Figure 2b). Under isotropic conditions, the simulated [11̅0] zone pattern reproduced the canonical σ phase intensity distribution. Selective enhancement of the temperature factor along the *c*-axis induced directional elongation of the motifs, amplifying the (330) reflection while suppressing the (002) reflection (middle panel of Figure 2b). Conversely, increasing the displacement within the *a–b* plane reversed this intensity trend (right panel of Figure 2b). These results demonstrate that anisotropic modulation of the Debye–Waller factor alone suffices to generate an orientation-dependent intensity redistribution, consistent with motif-level deformation within the unit cell.

**Figure 2:**
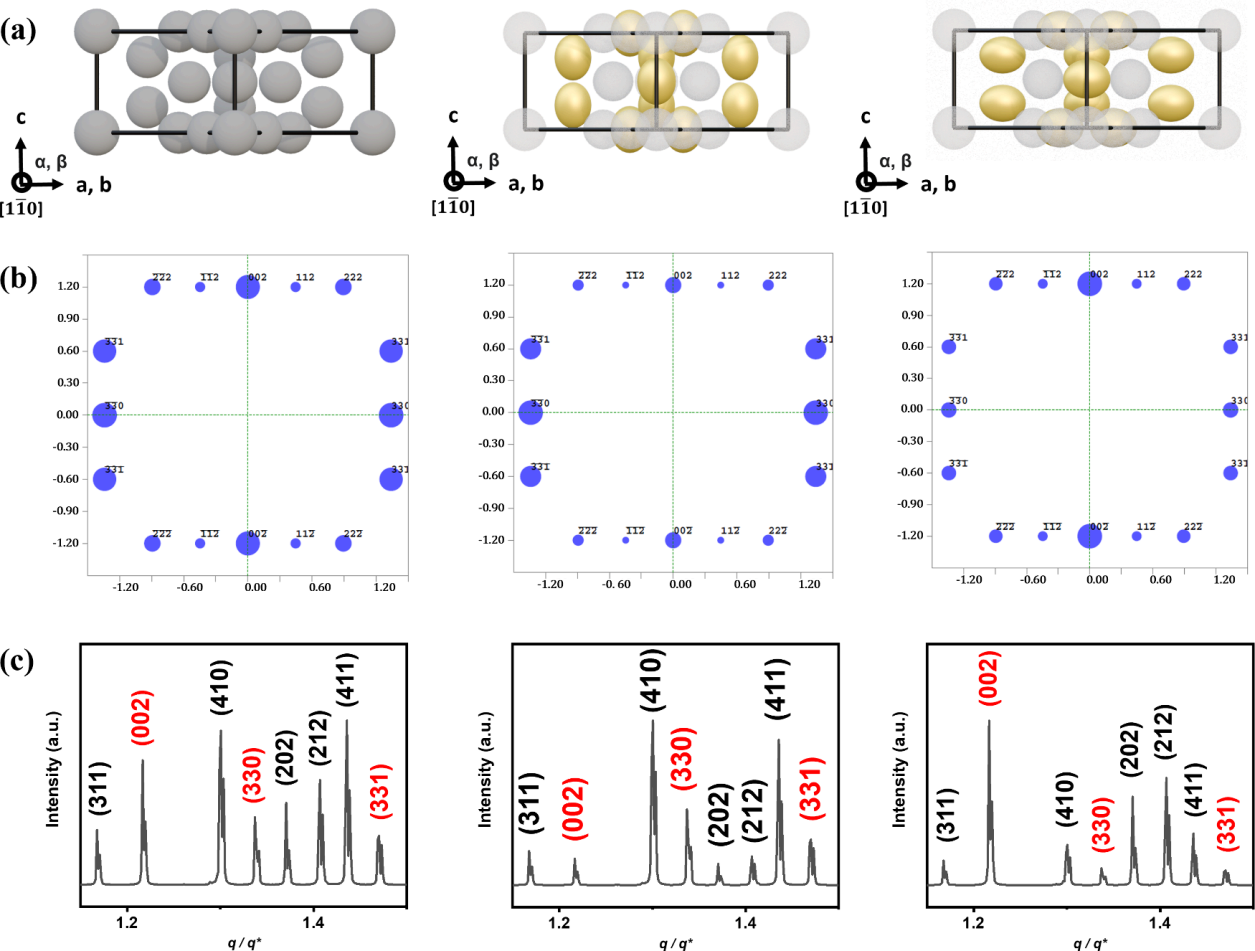
(a) Schematic representations of isotropic and anisotropic σ phase models, where isotropic (left) and directionally biased temperature factors along the *c*-axis (middle) or within the *a–b* plane (right) were applied. (b) Simulated [11̅0] zone axis diffraction patterns corresponding to the three models in panel a, showing how isotropic (left) and anisotropic temperature factors applied along the *c*-axis (middle) or within the *a–b* plane (right) influence the distribution and symmetry of reflection intensities. (c) Simulated powder diffraction patterns corresponding to the same three models in panel a, showing how directional modulation of the temperature factor alters the overall diffraction intensity distribution under isotropic and anisotropic conditions. q* represents the *q* value corresponding to the (310) reflection.

To further assess whether this anisotropy-driven scattering response persists under powder averaging conditions, complementary powder XRD simulations were conducted in Materials Studio, focusing on the (002), (330), and (331) reflections. Systematic variation of the temperature factor reproduced the same intensity evolution trends observed in the zone axis simulations and, critically, matched the experimental diffraction profiles of the AD σ phase with high fidelity, as shown in Figure 2c. The excellent agreement across theoretical formulation (eq 3), single-zone, and powder simulations, together with experimental diffraction, provides compelling evidence that chain-length asymmetry endows AD micelles with intrinsic anisotropy, enabling Debye–Waller-mediated deformation as a structural degree of freedom that dictates FK lattice symmetry.

Microbeam XRD revealed that the σ phase of the AD is composed of deformed micelles exhibiting grain-dependent random orientations. This observation indicates that the atypical diffraction:intensity ratios originate from motif-level anisotropy rather than lattice distortion. The orientational dispersion, however, suggests that the initially anisotropic σ phase may represent a metastable configuration.

To probe this metastability, we tracked the structural evolution of AD over time using SAXS measurements with a beam size of 400 μm × 200 μm, which integrated the scattering contributions from multiple σ phase grains. Freshly assembled samples displayed a well-defined σ phase diffraction pattern (Figure 3a). After 1 week, selective enhancement of the (410) reflection was detected, which further intensified in the second week and ultimately resulted in a complete σ-to-lamellar transformation after 3 weeks.

**Figure 3:**
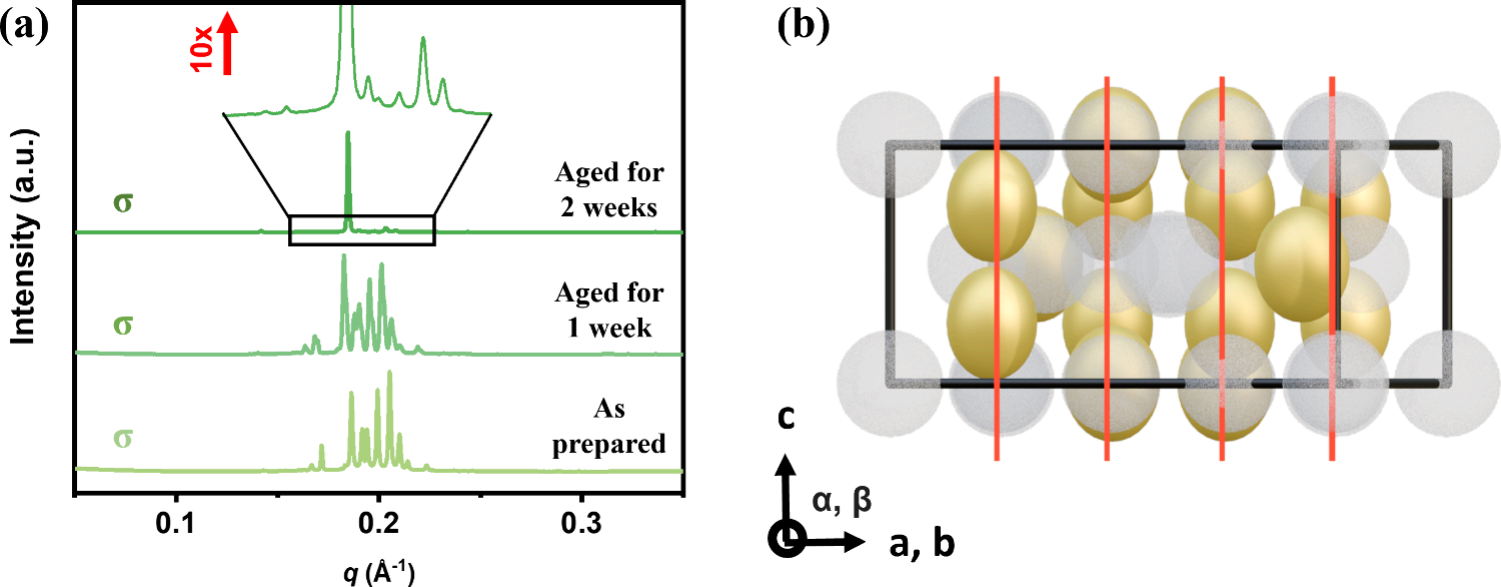
(a) Time evolution of SAXS patterns of AD σ phase assemblies, showing the gradual intensification of the (410) reflection over 2 weeks. (b) Geometric mapping of anisotropic motifs within the σ phase, illustrating the preferential alignment of ellipsoidal motifs along the (410) crystallographic planes, which accounts for the selective amplification of the (410) reflection. The red lines in the map represent the (410) crystallographic planes.

Literature analyses of polyhedral packing in the σ phase suggest that CN15 sites possess the largest free volume and are thus the most prone to ellipsoidal deformation, followed by CN14, while CN12 remains nearly spherical.^41^ By assigning the ellipsoidal shape to only CN15 and half of the CN14 motifs (ellipsoid fraction of ≈0.4), geometric mapping reveals that these anisotropic motifs preferentially align along the (410) crystallographic planes, as shown in Figure 3b. This spatial correlation is consistent with the selective amplification of the (410) reflection observed experimentally. Diffraction simulations incorporating temperature factor modulation (Figure S10) reproduce the observed intensity evolution, validating that orientation-selective scattering arises from anisotropic micellar deformation encoded in the Debye–Waller term.

Collectively, these results suggest that the σ phase of the AD initially forms as a mosaic of orientationally frustrated anisotropic motifs distributed among the grains. Upon aging, the motifs gradually reorient along the *c*-axis within the (410) planes to minimize the configurational free energy. This self-organized micellar alignment lowers the kinetic barrier for lattice rearrangement and serves as a thermodynamic precursor to the σ-to-lamellar transition. In this framework, motif anisotropy is not merely a passive source of diffraction distortion; it acts as a structural lever that dictates the kinetic accessibility of phase transitions in soft FK assemblies.

The discovery that AD micelles constitute the first supramolecular motif within the FK lattice capable of deformation prompted us to explore whether this deformability could be deliberately modulated. Because the anisotropy of AD originates from chain-length asymmetry within its hydrophobic shell, we reasoned that incorporating hydrophobic guest molecules might compensate for this asymmetry and, thereby, attenuate the deformation capacity of the micelles.

To examine this hypothesis, dodecane (C_12_; *T*_b_ = 216 °C) was selected as a hydrophobic guest and coassembled with the AD. As shown in Figure 4a, an AD:C_12_ ratio of 1:1 produced phase behavior comparable to that of a neat AD. When the C_12_ content was increased to a 1:3 ratio, however, a σ-to-bcc phase transition was detected near *T*_i_ (Figure 4b). Further changing the C_12_ ratio to 1:5 and 1:7 suppressed symmetry breaking entirely, yielding a stable bcc phase at room temperature (Figure 4c,d and Figure S11). This trend is summarized in Figure 4e, where the bar chart clearly shows the progressive suppression of the σ phase and the corresponding stabilization of the bcc phase with an increase in C_12_ content. Because bcc packing lacks both anisotropy and symmetry breaking, the appearance of a σ → bcc pathway suggests that C_12_ incorporation compensates for the chain-length asymmetry of the AD, effectively diminishing micellar deformability and mitigating the frustration-driven stabilization of the σ phase. In contrast, the SD, which inherently lacks micellar deformability, exhibited no such phase transition upon addition of C_12_ (Figure S12).

**Figure 4:**
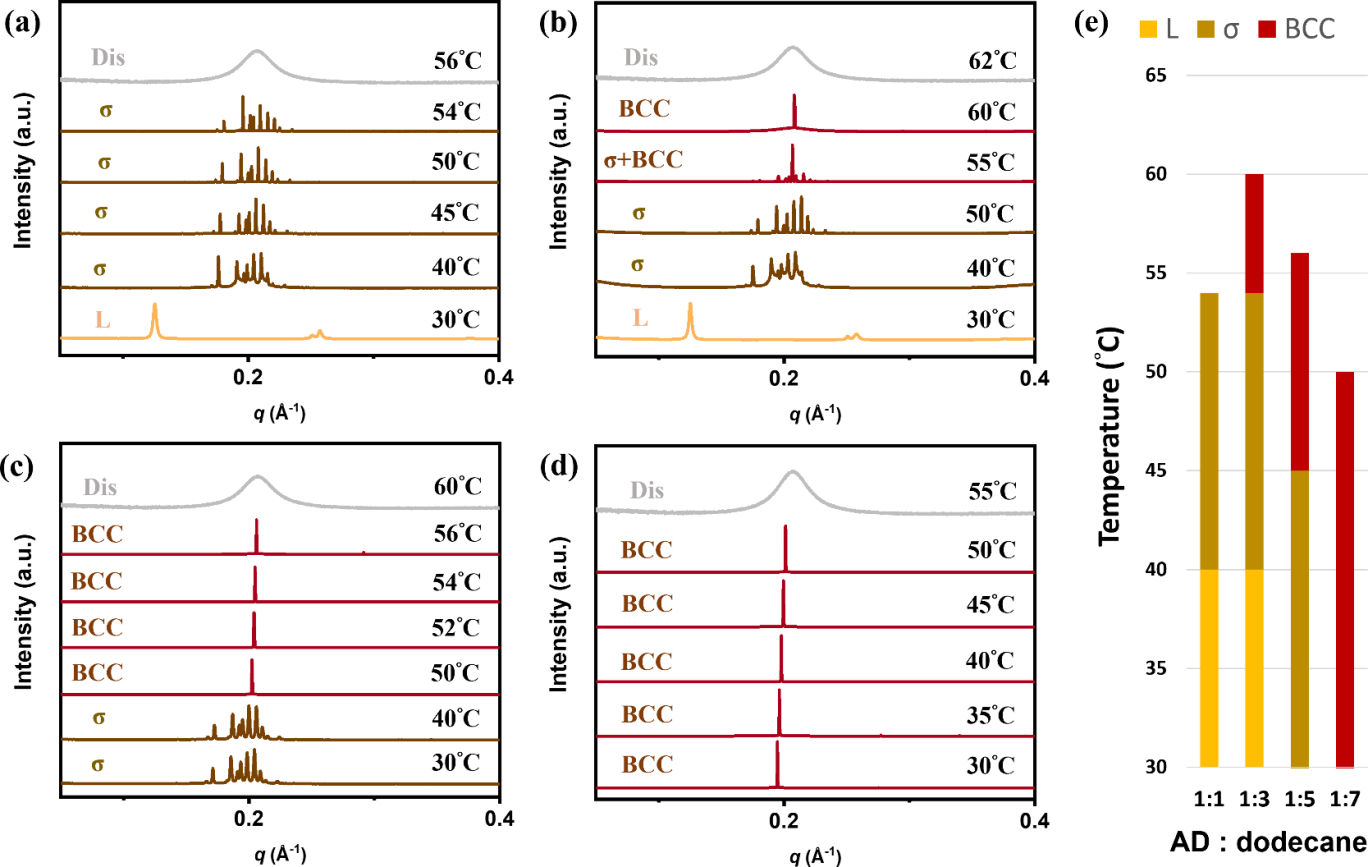
Temperature-dependent SAXS profiles of the AD/C_12_ mixtures at ratios of (a) 1:1, (b) 1:3, (c) 1:5, and (d) 1:7. (e) Phase behaviors of the AD/C_12_ mixtures (AD:C_12_ ratios of 1:1–1:7).

In summary, this study demonstrates that chain-length asymmetry imparts intrinsic deformability to supramolecular micelles formed from dendritic amphiphiles, thereby enabling anisotropic stabilization of the soft FK σ phase. Distinct from conventional symmetry breaking driven by size or shape disparity, this deformability manifests as directional modulation within the lattice, producing a domain-specific redistribution of diffraction intensity. The anisotropic micelles, initially orientationally frustrated, gradually reorganize to mediate the σ-to-lamellar transition, establishing a mechanistic link between motif anisotropy and kinetic phase evolution.

Crucially, we further show that this deformability can be selectively suppressed by incorporating hydrophobic guest molecules that compensate for chain-length asymmetry, thereby recovering isotropic bcc packing. Such “on–off” control of micellar deformability unveils a new degree of freedom in soft FK assemblies and highlights deformability as a programmable parameter for designing adaptive and reconfigurable supramolecular architectures.

## Supplementary Material





## Notes

Synthetic procedures; ^1^H NMR, ^13^C NMR, and mass spectra of the AD; structural characterization, including unit cell parameters, Miller indices, and SAXS patterns of the FK σ phase of the AD and SD; instrumentation of the analytical experiments; simulation methods; and temperature-dependent SAXS profiles of the SD/C_12_ mixtures (PDF)
Transparent Peer Review report available (PDF)
