# Supplementary material for: On and Off Deformability of Supramolecular Micelles in the Soft Frank–Kasper σ Phase
Source: J Phys Chem Lett. 2026 Mar 20;17(13):3947–53. doi: 10.1021/acs.jpclett.6c00287 (PMC13051438; doi:10.1021/acs.jpclett.6c00287)
Supplement: Supplementary file 1 [file jz6c00287_si_001.pdf]

Supporting Information

On and Off Deformability of Supramolecular Micelles in the Soft Frank-Kasper σ Phase.

Shih-Yong Chen[a], Chin-Hong Goh[a], Mayumi Egashira[a], Chun-Yu Chen[b], Jhih-Min Lin[b]*, Chien-Lung Wang[a]*

[a]Department of Chemistry, National Taiwan University, No. 1, Sec. 4, Roosevelt Rd, Taipei 10617, Taiwan

[b]National Synchrotron Radiation Research Center, 101 Hsin-Ann Road, Hsinchu 30076, Taiwan

All authors have given approval to the final version of the manuscript.

*** Corresponding Author**

Email: kclwang@ntu.edu.tw

*** Corresponding Author**

Email: lin.jm@nsrrc.org.tw

Table of Contents

Experimental Section 1
1. Synthetic Procedures of the Asymmetric Dendron (AD)..................................1
2. Analytical Experiments 2
Characteristic Section...4
1. NMR and MS Spectra of Products 4
2. Structural Characterization of the AD and SD 7
References 12

Experimental Section

1. Synthetic Procedures of the Asymmetric Dendron (AD)

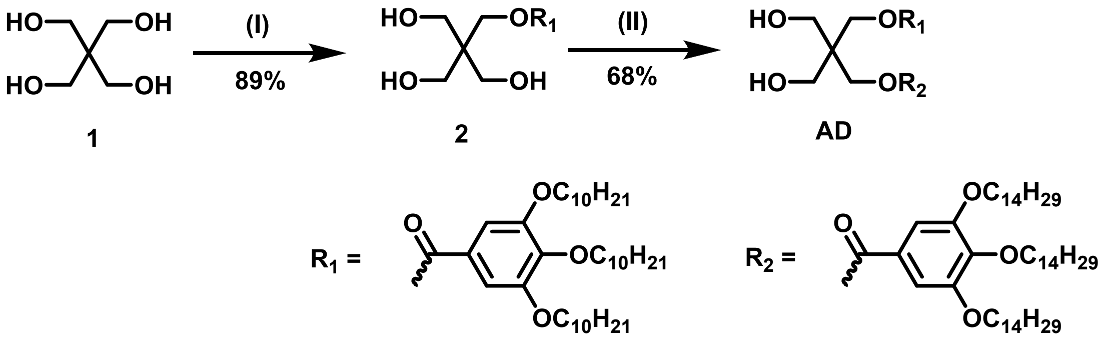

Scheme S1. The synthetic route of **AD**. (I) **3,4,5-Tris(decyloxy)benzoic acid,** 4-(Dimethylamino)pyridine (DMAP), 1-(3-Dimethylaminopropyl)-3-ethylcarbodiimide (EDC), DMF+DCM, 25°C, 24 h ; (II) **3,4,5-Tris(tetradecyloxy)benzoic acid,** 4-(Dimethylamino)pyridine (DMAP), 1-(3-Dimethylaminopropyl)-3-ethylcarbodiimide (EDC), DCM, 25 °C, 24 h

Scheme S1 shows the synthetic route of the **AD**. The **AD** was synthesized by sequentially connecting **3,4,5-Tris(decyloxy)benzoic acid** and **3,4,5-Tris(tetradecyloxy)benzoic acid** to a pentaerythritol core *via* Steglich esterification. The **3,4,5-Tris(decyloxy)benzoic acid** and **3,4,5-Tris(tetradecyloxy)benzoic acid** were synthesized according to the literature.[1, 2] First, in order to get single substitution product as much as possible, we added 5 equiv. of pentaerythritol to **3,4,5-Tris(decyloxy)benzoic acid** to synthesize **compound 2**. The ^{1}H-NMR, ^{13}C-NMR and ESI-MS spectra of **compound 2** are shown in **Figure S1 – S3**. **^{1}H NMR (400 MHz, Chloroform-*d*)** δ 7.24 (s, 2H), 4.46 (s, 2H), 4.01 (m, 6H), 3.73 (d, 6H), 2.52 (s, 3H), 1.81 (m, 6H), 1.48 (m, 6H), 1.27 (s, 36H), 0.88 (t, 9H). ^{13}C NMR (101 MHz, $CDCl_3$) δ 167.65, 153.11, 143.27, 123.94, 108.52, 77.48, 77.16, 76.84, 73.75, 69.50, 63.89, 63.46,

45.97, 32.06, 30.48, 29.78, 29.49, 26.24, 22.83, 14.24. ESI-MS $[M+H]^+$ = 709.5613 for $C_{42}H_{76}O_8$. Afterward, the reaction of 3 equiv. of **compound 2** and 1 equiv. of **3,4,5-Tris(tetradecyloxy)benzoic acid** *via* Steglich esterification gave the final compound **AD**. The ^{1}H-NMR, ^{13}C-NMR and ESI-MS spectra of **AD** are shown in **Figure S4 – S6**. **^{1}H NMR (400 MHz, Chloroform-*d*)** δ 7.23 (s, 4H), 4.47 (s, 4H), 4.04 – 3.96 (m, 12H), 3.71 (d, 4H), 3.01 (t, 2H), 1.80 (m, 12H), 1.47 (m, 12H), 1.26 (m, 98H), 0.88 (t, 18H). ^{13}C NMR (101 MHz, $CDCl_3$) δ 167.04, 152.85, 142.97, 123.56, 108.16, 77.22, 76.90, 76.58, 73.47, 69.17, 62.97, 62.67, 45.92, 31.82, 30.23, 29.61, 29.24, 26.00, 22.58, 13.99. MALDI-TOF $[M+Na]^+$ = 1473.2111 for $C_{91}H_{164}O_{12}$.

2. Analytical Experiments

Materials and Instrumentation: ^{1}H and ^{13}C nuclear magnetic resonance (NMR) spectra were obtained using a Bruker AVIII HD 400 NMR spectrometer (400 MHz for ^{1}H and 101 MHz for ^{13}C) with $CDCl_3$ as the deuterated solvent. Measurements were conducted at room temperature to determine the molecular structures. The Matrix-assisted laser desorption/ionization mass spectrometry (MALDI-MS) and the electrospray ionization mass spectrometry (ESI-MS) were performed on a time-of-flight (TOF) instrument to determine the molecular weight of the synthetic compounds. **Small/Wide-angle X-ray scattering (S/WAXS):** Small- and wide-angle X-ray scattering measurements were performed on the TPS 13A beamline and TPS 25A1 at the National Synchrotron Radiation Research Center (NSRRC), Taiwan. TPS 13A is a SWAXS beamline, and the measurements were operated with an X-ray wavelength of 0.8265 Å and a beam focus size of 400 × 200 μm². The scattering vector (q) ranged from 0.004 to 0.45 Å^{-1} for SAXS and 0.4 to 1.7 Å^{-1} for WAXS. TPS 25A1 is a

microbeam X-ray scattering beamline operated at 5.5–20 keV. The experiments were conducted with an X-ray wavelength of 0.8265 Å and a beam focus size of 5 × 5 μm². The scattering vector (q) ranged from 0.05 to 0.5 $Å^{-1}$ for SAXS. Approximately 1 mg of each sample was wrapped in two layers of heat-resistant Kapton tape for measurement.

Simulation Methods (Cerius² / Materials Studio): The σ-phase crystallographic model was constructed in Cerius² / Materials Studio by following the prototype information in the Encyclopedia of Crystallographic Prototypes. Specifically, we used the σ-phase (tetragonal, space group $P4_2/mnm$, No. 136; σ-Fe–Cr prototype) and imported the fractional atomic coordinates for each crystallographic site as reported in reference.[3]

To isolate the effect of thermal/disorder displacements on diffraction intensities, atomic displacement parameters (ADPs) were controlled globally:

1. Isotropic σ:

   We selected Global Isotropic displacement in the Diffraction module and set the isotropic parameter to

$$U_{\text{iso}} = 0.10\grave{A}^2$$

This setting constrains thermal motion to be direction-independent for all sites, modeling the isotropic σ phase formed by SD micelles.

2. Anisotropic σ:

   We then enabled Global Anisotropic displacement and prescribed diagonal components in the crystallographic frame as

Case A: $U_a = U_b = 0.10\grave{A}^2$,$U_c = 0.20\grave{A}^2$;

Case B (swapped): $U_a = U_b = 0.20\ \grave{A}^2$,$U_c = 0.10\ \grave{A}^2$. Case A emphasizes displacements along the c axis relative to the ab plane (i.e., $U_c = 2U_a$), whereas Case

B emphasizes the ab plane relative to c, thereby bracketing the anisotropic behavior characteristic of AD micelles.

Characteristic Section

1. NMR and MS Spectra of Products

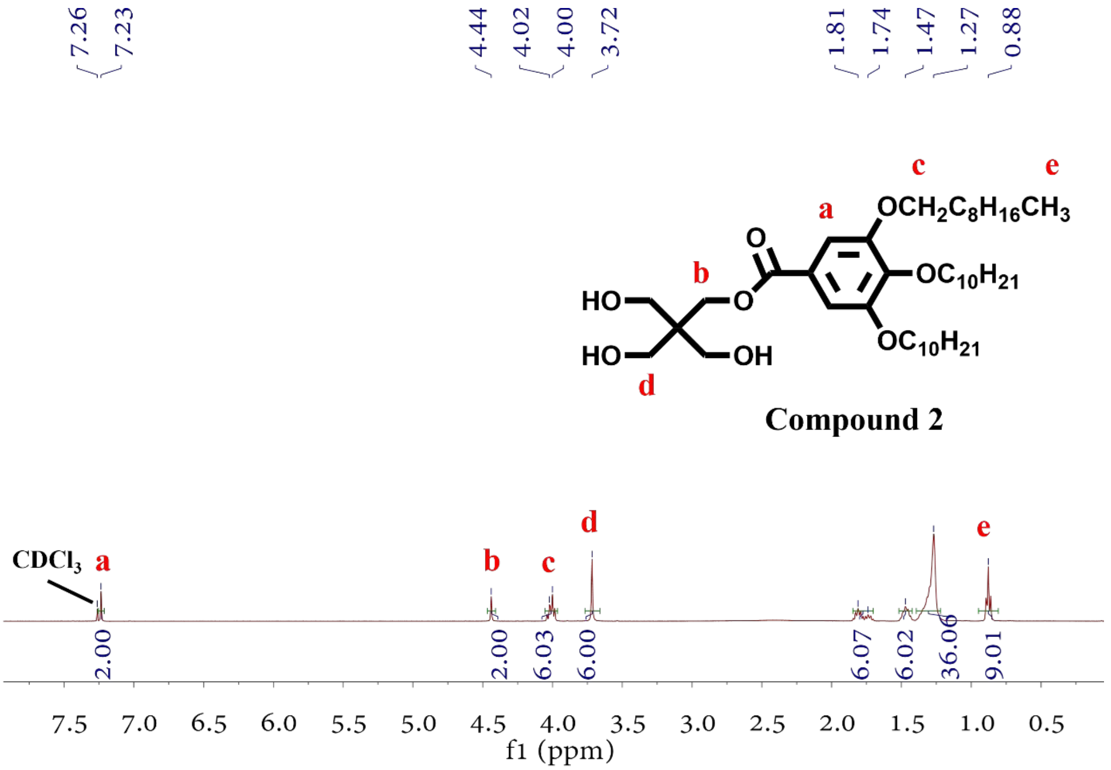

Figure S1. ^1H NMR spectra of **compound 2** (400 MHz in $CDCl_3$).

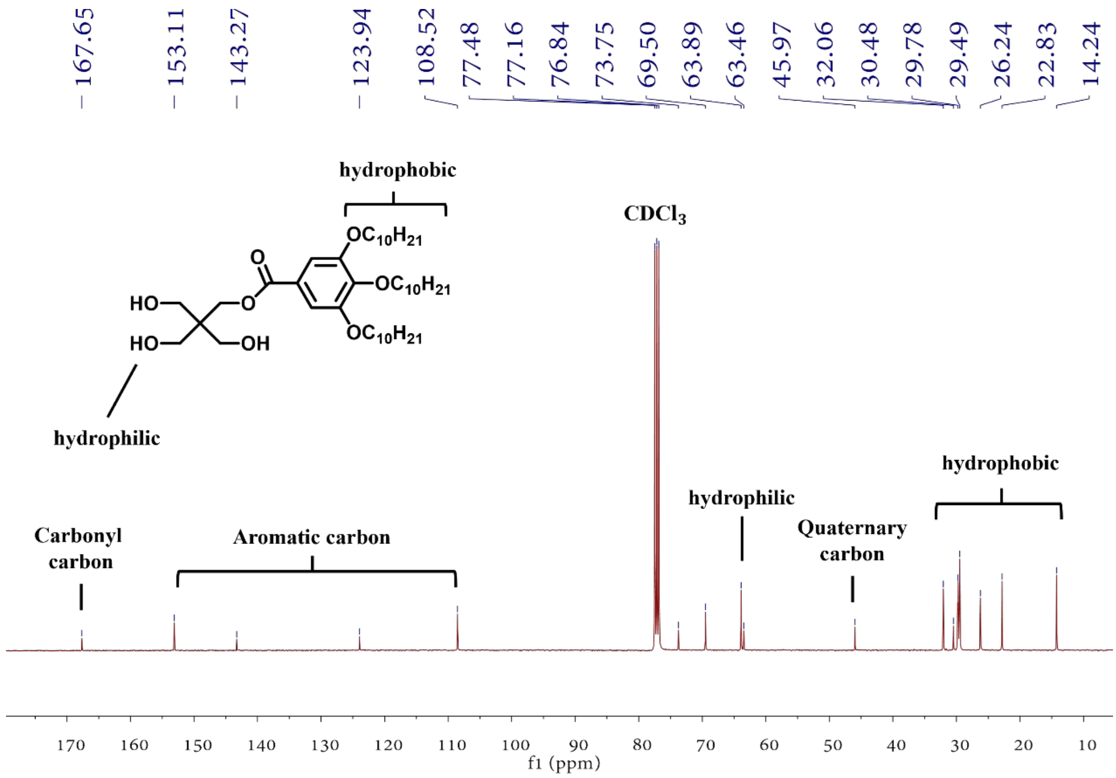

Figure S2. ^{13}C NMR spectra of **compound 2** (101 MHz in $CDCl_3$).

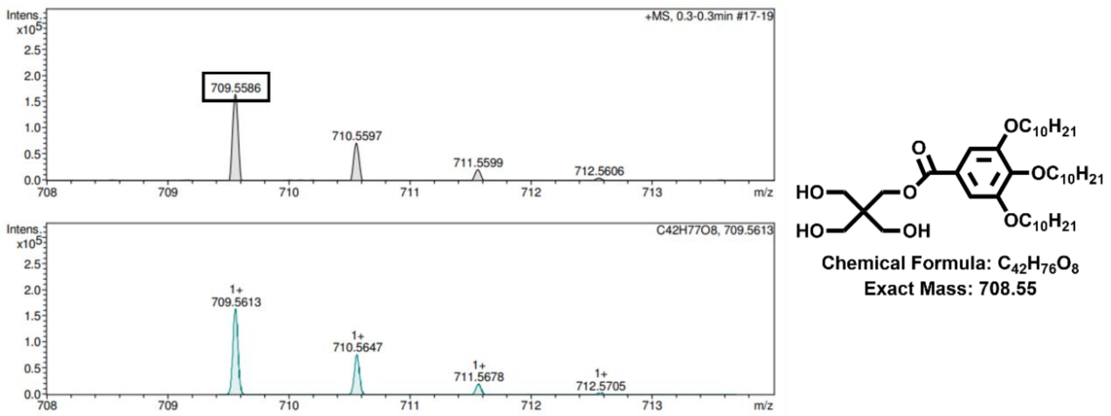

Figure S3. ESI-MS spectra of **compound 2**.

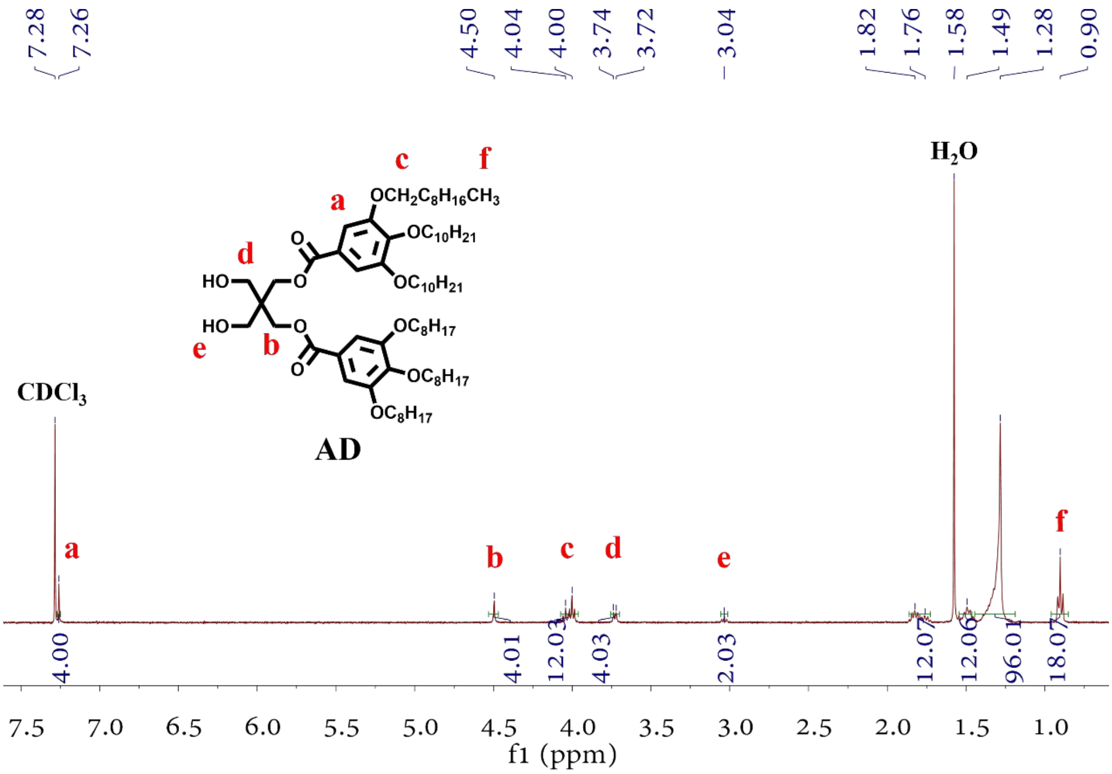

Figure S4. ^{1}H NMR spectra of **AD** (400 MHz in $CDCl_3$).

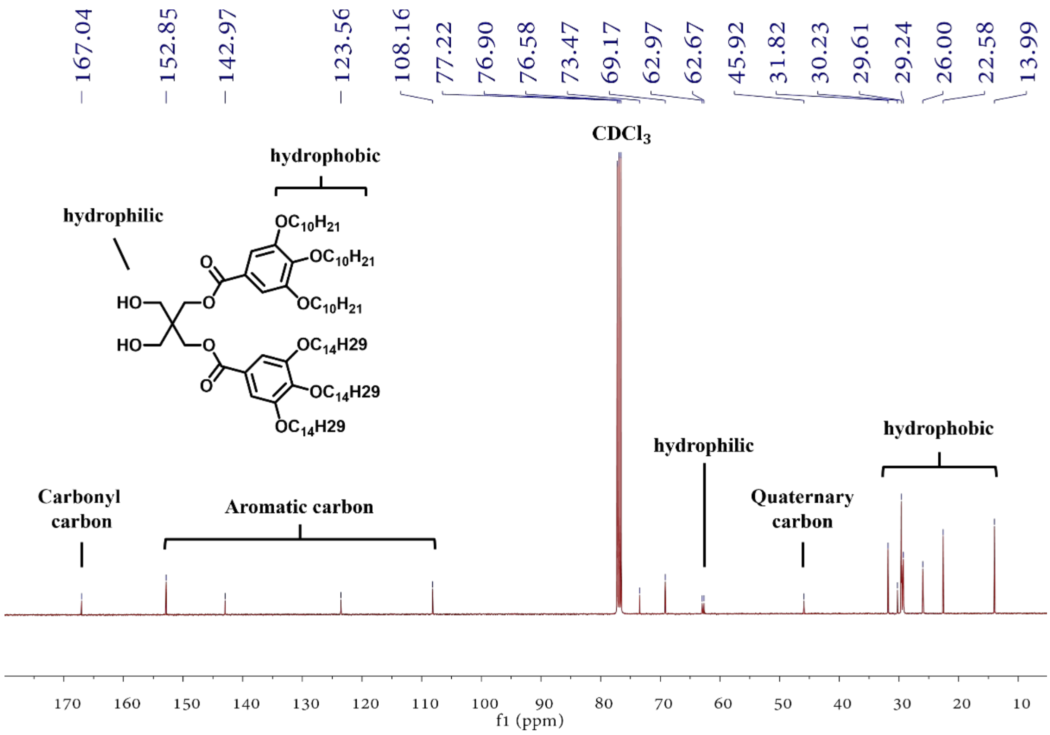

Figure S5. ^{13}C NMR spectra of **AD** (101 MHz in $CDCl_3$).

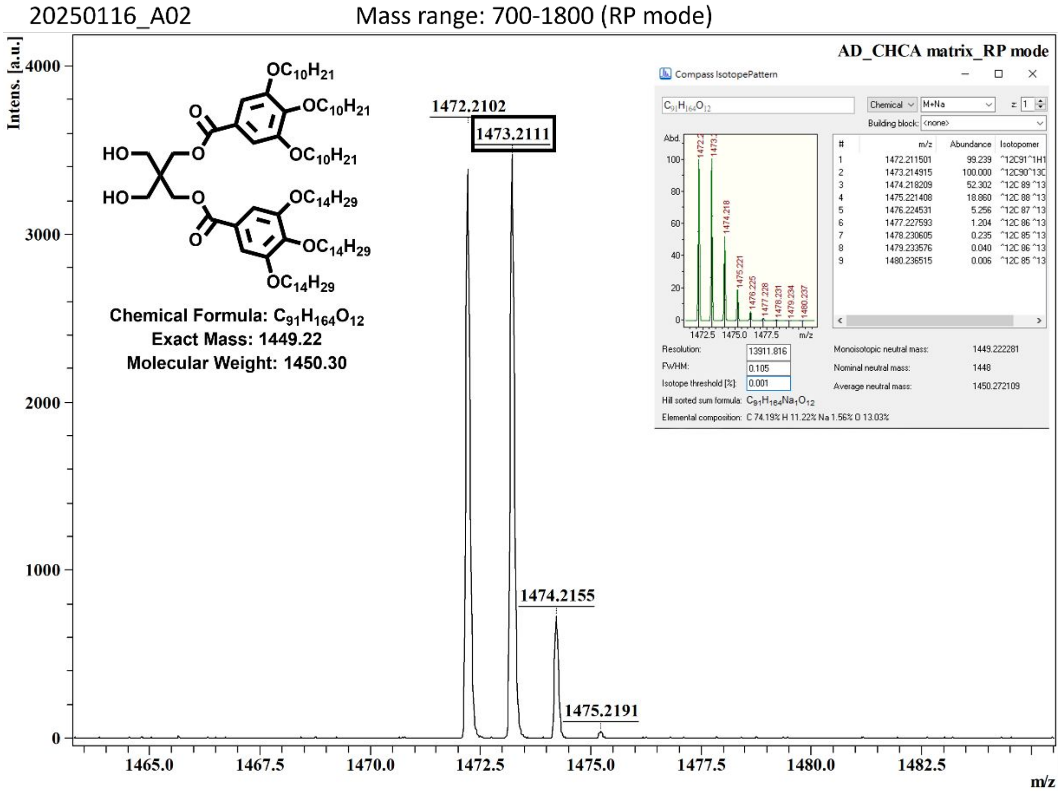

Figure S6. MALDI-TOF spectra of the [AD+Na$^+$].

2. Structural Characterization of the AD and SD

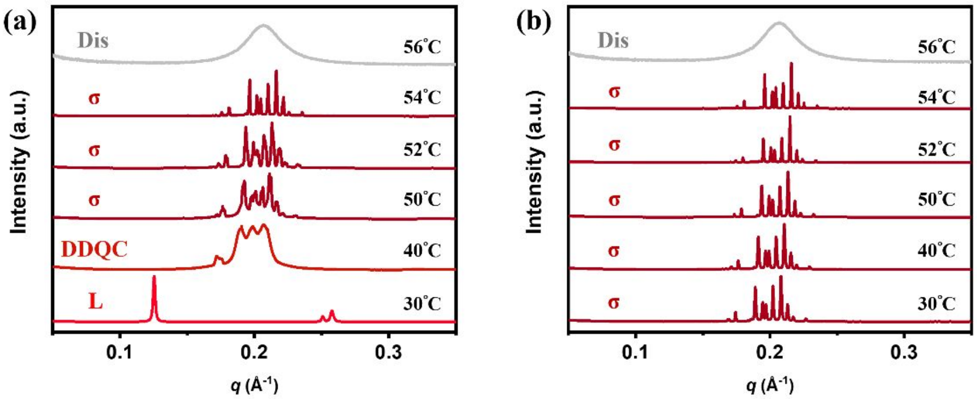

Figure S7. The temperature-dependent SAXS profiles of AD in (a) 1st heating, and (b) 2nd heating scans.

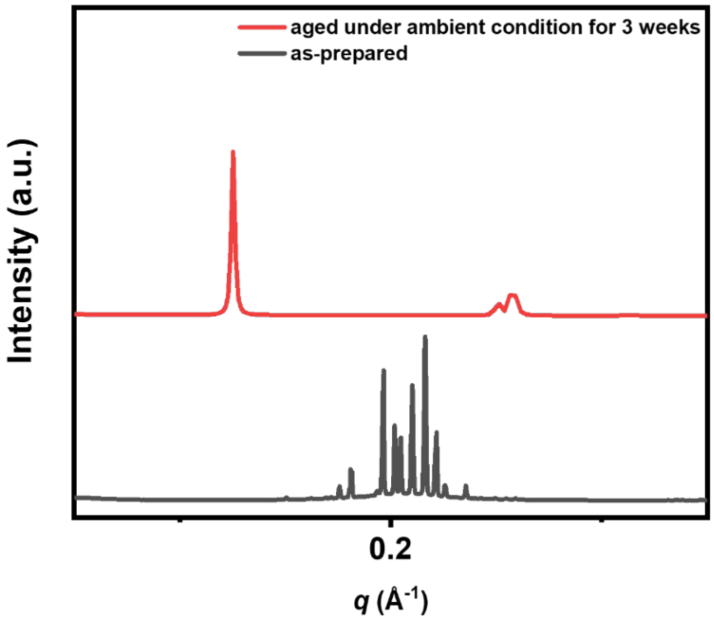

Figure S8. The SAXS patterns of the as-prepared σ phase of **AD** and the same sample after aged under ambient condition for three weeks.

$$a = b = 3\sqrt{2} \times d_{330} \quad \text{(Eq. S1)}$$

$$c = 2 \times d_{002} \quad \text{(Eq. S2)}$$

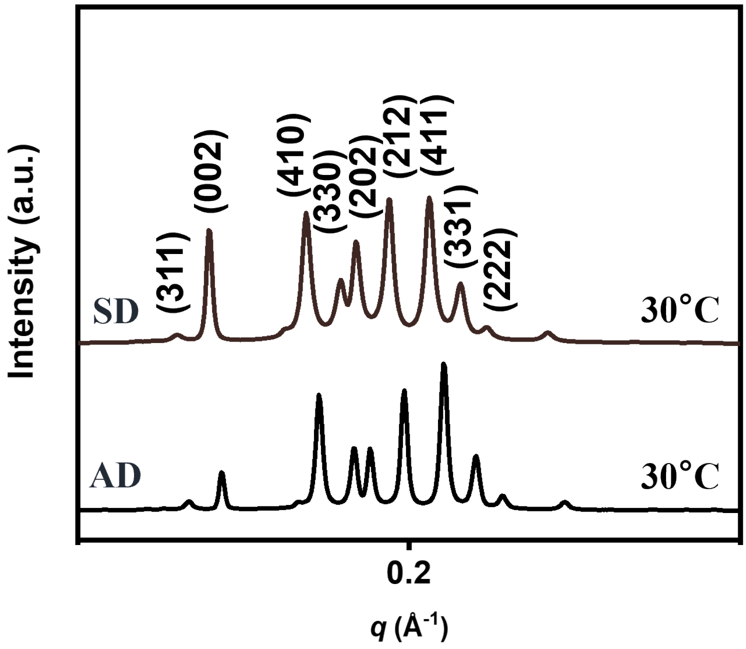

Figure S9. The SAXS profile of **SD** and **AD** in the σ phase at 30°C, along with peak

indexing.

Table S1. Comparison of the unit cell parameters between the σ phase of **AD** and **SD** at RT.

	**AD**	**SD**
**a, b (Å^{-1})**	**139.16**	**140.56**
**c (Å^{-1})**	**73.22**	**73.95**

Table S2. List of the calculated and observed q for the σ phase of **AD** at 30˚C.

**Miller indices (hkl)**	q_{calc} **(Å^{-1})**	q_{calc} **ratio**	q_{obs} **(Å^{-1})**	q_{obs} **ratio**	$\Delta q/q_{calc}$ **(%)**
(310)	0.1456	1.00	0.1464	1.00	0.55
(221)	0.1570	1.08	0.1571	1.07	0.06
(301)	0.1636	1.12	0.1634	1.12	-0.12
(320)	0.1660	1.14	0.1667	1.14	0.42
(311)	0.1699	1.17	0.1700	1.16	0.06
(002)	0.1752	1.20	0.1752	1.20	0
(410)	0.1899	1.30	0.1896	1.30	-0.16
(330)	0.1954	1.34	0.1954	1.33	0
(202)	0.1979	1.36	0.1993	1.36	0.71
(212)	0.2032	1.39	0.2033	1.39	0.05
(411)	0.2091	1.44	0.2096	1.43	0.24
(331)	0.2141	1.47	0.2137	1.46	-0.19
(312)	0.2278	1.56	0.2290	1.56	0.53

$(h_1\, k_1\, l_1) \times (h_2\, k_2\, l_2) = (\mathrm{u\,v\,w})$ (Eq. S3)

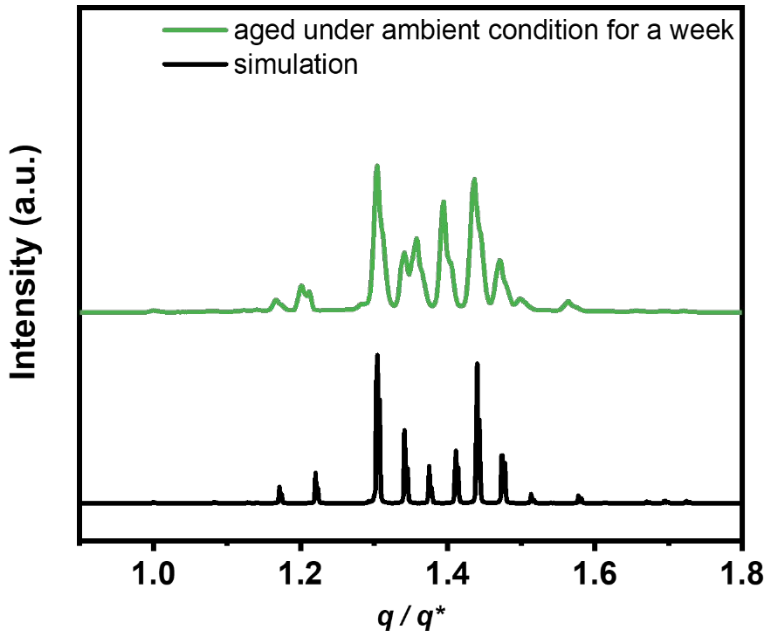

Figure S10. The simulated SAXS pattern with an enhanced c-axis temperature factor compared with that of the **AD** sample after one week of preparation. q^* represents the (310) peak position.

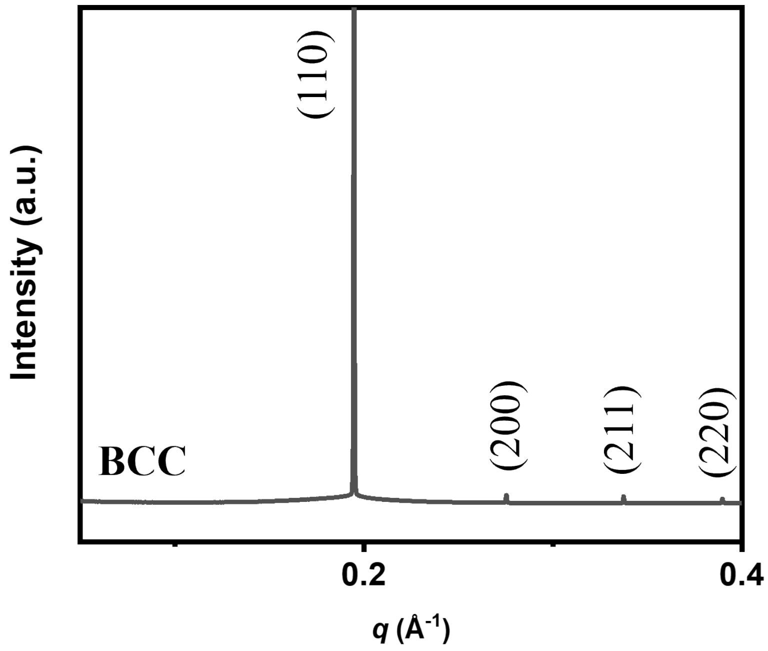

Figure S11. The SAXS profile of the AD:C12 mixtures (1:7) at room temperature.

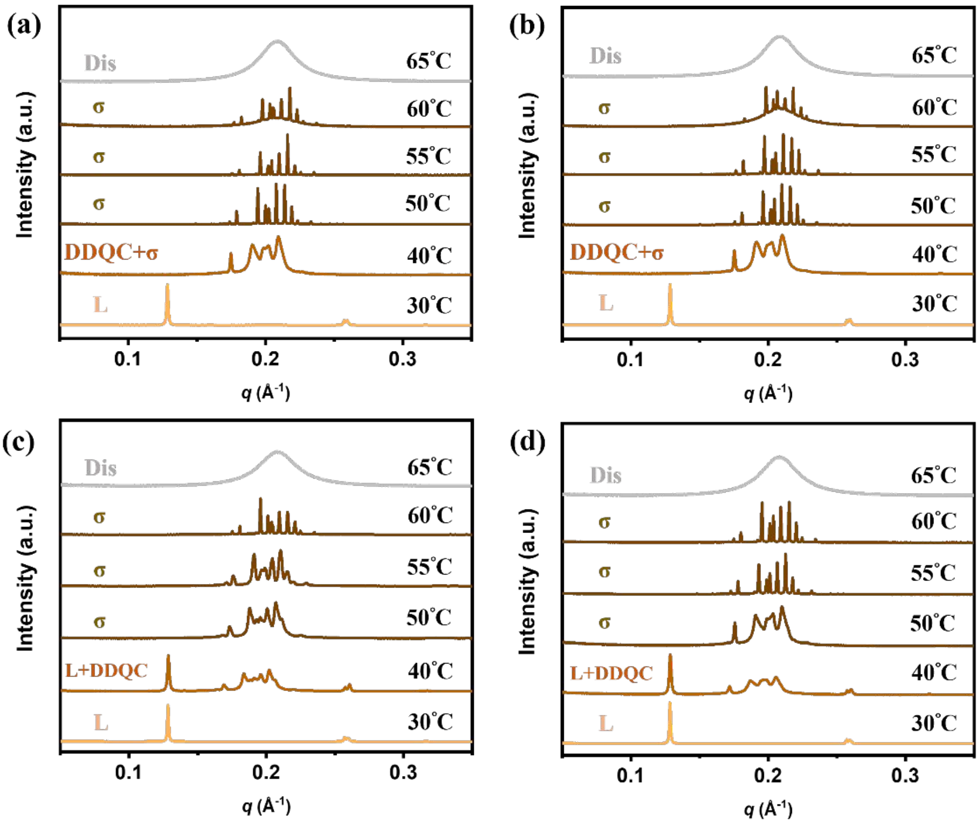

Figure S12. Temperature-dependent SAXS profiles of the SD:C_{12} mixtures at a (a) 1:1 (b) 1:3 (c) 1:5 (d) 1:7 ratio.

References

1. V. Percec, C. H. Ahn, T. K. Bera, G. Ungar, D. J. Yeardley, *Chem. Eur. J.*, 1999, **5**, 1070-1083.
2. V. Percec, M. N. Holerca, S. Uchida, W. D. Cho, G. Ungar, Y. Lee, D. J. Yeardley, *Chem. Eur. J.*, 2002, **8**, 1106-1117.
3. H. Yakel, *Acta Crystallogr., Sect. B:Struct. Sci.* 1983, **39**, 20-28.
