# Supplementary material for: On and Off Deformability of Supramolecular Micelles in the Soft Frank–Kasper σ Phase
Source: J Phys Chem Lett. 2026 Mar 20;17(13):3947–53. doi: 10.1021/acs.jpclett.6c00287 (PMC13051438; doi:10.1021/acs.jpclett.6c00287)
Supplement: Supplementary file 2 [file jz6c00287_si_002.pdf]

jz-2026-00287u.R1

Name: Peer Review Information for "On and Off Deformability of Supramolecular Micelles in the Soft Frank-Kasper σ Phase."

First Round of Reviewer Comments

Reviewer: 1

Comments to the Author

The manuscript “On and Off Deformability of Supramolecular Micelles in the Soft Frank–Kasper σ Phase” by Chen and co-authors reports the synthesis of asymmetric dendrons and the characterization of their self-assembled micellar aggregates forming Frank–Kasper-type structures. The work proposes an elegant strategy to modulate soft-matter organization through molecular asymmetry, with the aim of establishing a design principle for adaptive and hierarchically complex assemblies.

The experimental toolbox employed—temperature-dependent SAXS, WAXS, mass spectrometry, and NMR spectroscopy—is in principle appropriate for the investigation of such systems, which are of clear interest to the community working on Frank–Kasper phases and complex self-assembly.

However, I have significant concerns regarding the quality and reliability of the NMR characterization of the dendrons. This represents a critical weakness of the manuscript and should be addressed before the work can be considered robust.

NMR Characterization

The NMR spectra provided in the Supporting Information are not sufficient to unambiguously confirm the proposed dendron structures. In particular, the integrated areas reported in the ¹H NMR spectra show significant deviations from the expected values, often exceeding what could reasonably be attributed to experimental uncertainty. In several cases, the selected integration regions appear artificially restricted,

seemingly chosen to approximate the expected proton counts rather than reflecting the true signal envelopes.

Moreover, essential experimental details regarding the NMR measurements are missing. It is not specified whether the ¹H NMR experiments were performed under quantitative conditions. No information is provided regarding T_{$_{1}$} relaxation times, repetition delays, or whether a delay of at least five times the longest T_{$_{1}$} was employed, as required for reliable quantitative integration. Without these details, the reported integrals cannot be considered trustworthy.

Specific Issues in Figure S1 (Compound 2)

In Figure S1, the authors appear to normalize the integrals to the aromatic signal at approximately 7.2 ppm. A signal at ~4.5 ppm is observed having integral equal to 2.0: the authors should assign this signal.

Between 3.5 and 4.25 ppm, two signals are reported with integrals of 6.35 and 6.12, whereas values close to 6.0 would be expected. The systematic overestimation of these integrals strongly suggests insufficient relaxation of the reference signal, consistent with an inadequately short relaxation delay.

A (relatively broad) signal at 2.5 ppm, with an integral of 3.0, is reported but not assigned - The authors should clarify whether this signal originates from OH groups or from another structural motif.

Additional resonances at approximately 1.75 and 1.5 ppm also display integrals that are clearly too large, whereas values around 6.0 would be expected.

At 1.25 ppm, a large aliphatic signal is observed. Although the expected integral should be 36, the reported value is 36.77. More importantly, the chosen integration region is clearly too narrow to capture the full signal envelope, making the reported value unreliable. This gives the impression that the integration window was adjusted to match the expected number rather than determined objectively.

Finally, the methyl signal at approximately 0.8 ppm is reported with an integral of 9.12, again significantly larger than the expected value of ~9.0.

Taken together, these issues raise serious concerns about the level of control over the synthesis and characterization of the precursor (compound 2). A more rigorous and transparently quantitative NMR characterization is required.

Asymmetric Dendron (Figures S4–S5)

Similar issues are present in the ¹H NMR characterization of the asymmetric dendron shown in Figures S4–S5. Although some signal assignments are provided, the same problems with inconsistent and systematically overestimated integrals persist. The reported positive deviations are significantly larger than expected for properly acquired quantitative ¹H NMR spectra.

The authors should provide new NMR data acquired under well-defined quantitative conditions and include a clearer discussion of signal assignments and integrations to convincingly validate the proposed dendron structures.

In conclusion, a more rigorous NMR characterization would significantly strengthen the manuscript. At present, the NMR characterization is not sufficiently robust to fully support the otherwise high-quality solid-state and scattering-based analysis of the Frank–Kasper phases.

Reviewer: 2

Comments to the Author

In this manuscript, by demonstrating that micelles within a complex spherical phase can adopt systematic, site-dependent anisotropic shapes, the authors introduce a compelling stabilization paradigm in which micellar deformability actively sculpts the FK phase energy landscape. This work forms a conceptual bridge between sphericity-governed theories and anisotropy-enabled simulations and represents an important and innovative advance in the study of complex spherical phases. Accordingly, the reviewer finds that this manuscript achieves the originality and significance expected of publications in J. Phys. Chem. Lett.. Several clarifications and additional experiments, if feasible, would further strengthen the mechanistic interpretation. Detailed comments are provided below.

1. The manuscript focuses on one degree of asymmetry (C10/C14). Have more asymmetric combinations (e.g., C8/C16 or other extreme mismatches) been tested?

Such results would clarify whether: deformability increases monotonically with asymmetry, an upper limit exists beyond which FK stability is lost, or excessive asymmetry produces new phases. Addressing this point would significantly strengthen the mechanistic framework.

2. In Figure 4, the (410) reflection intensifies dramatically after two weeks, signaling substantial motif reorientation or deformation accumulation. Yet the simulations in Figure S9 reproduce only the one-week evolution. The reviewer requests clarification:

What additional structural reorganization occurs between week 1 and week 2 that is absent from the current model? A more explicit discussion would improve the alignment between experiment and simulation.

3. About the long-term stability of BCC at high C12 content, for mixtures with very high C12 content (1:7), is the resulting BCC phase thermodynamically stable? Does it persist upon long-term aging, or does it transform into σ or lamellar phases analogous to those observed in neat AD? Clarification would improve understanding of the phase diagram.

4. Cheng and co-workers (Nat. Mater. 2024, 23, 570–576) recently demonstrated unique soft μ and ϕ phase constructed with mesoatom. In the soft ϕ phase, a low fraction of mesoatoms was found to have aspect ratio of ca. 1.2. Although their system is non-amphiphilic and more rigid, lacking the on/off deformability of the present system, and the XRD structural characterization did not reveal the anisotropy of the mesoatoms. A brief acknowledgment would strengthen the manuscript.

Author's Response to Peer Review Comments:

Dear Editor,

Thank you for handling our manuscript " On and Off Deformability of Supramolecular Micelles in the Soft Frank-Kasper σ Phase." submitted to The Journal of Physical Chemistry Letters (Manuscript ID: jz-2026-00287u). The manuscript has been carefully

revised in response to the reviewers' constructive and thoughtful comments. The authors sincerely appreciate the time and effort invested by the editorial office and the reviewers, whose suggestions have helped improve the clarity, rigor, and overall quality of the manuscript.

We have addressed each comment in detail and revised the manuscript and the Electronic Supporting Information accordingly. All changes in the revised version are highlighted in red for ease of reference. Please find below our point-by-point responses to the reviewers' comments. We hope that the revised manuscript adequately addresses the reviewers' concerns and contributes more clearly to the intended scientific discussion. Thank you very much for your consideration.

REVIEWER REPORT(S):

Reviewer: 1

Comments:

1. *In Figure S1, the authors appear to normalize the integrals to the aromatic signal at approximately 7.2 ppm. A signal at ~4.5 ppm is observed having integral equal to 2.0: the authors should assign this signal.*

Between 3.5 and 4.25 ppm, two signals are reported with integrals of 6.35 and 6.12, whereas values close to 6.0 would be expected. The systematic overestimation of these integrals strongly suggests insufficient relaxation of the reference signal, consistent with an inadequately short relaxation delay.

A (relatively broad) signal at 2.5 ppm, with an integral of 3.0, is reported but not assigned - The authors should clarify whether this signal originates from OH groups or from another structural motif.

Additional resonances at approximately 1.75 and 1.5 ppm also display integrals that are clearly too large, whereas values around 6.0 would be expected.
At 1.25 ppm, a large aliphatic signal is observed. Although the expected integral should be 36, the reported value is 36.77. More importantly, the chosen integration region is clearly too narrow to capture the full signal envelope, making the reported value unreliable. This gives the impression that the integration window was adjusted to match the expected number rather than determined objectively.
Finally, the methyl signal at approximately 0.8 ppm is reported with an integral of 9.12, again significantly larger than the expected value of ~9.0.
Taken together, these issues raise serious concerns about the level of control over the synthesis and characterization of the precursor (compound 2). A more rigorous and transparently quantitative NMR characterization is required.

Response:
We thank the reviewer for highlighting the importance of sufficient relaxation in quantitative ^{1}H NMR measurements. In response to this comment, we have reacquired the ^{1}H NMR spectrum of compound **2** under quantitative conditions using a relaxation delay (D_1) of 15 s. For typical small- to medium-sized organic molecules in $CDCl_3$, aromatic proton T_1 values are generally on the order of a few seconds. A 15 s relaxation delay therefore exceeds five times the expected longest T_1 and ensures complete relaxation prior to each pulse, providing reliable and quantitative integral values.
Regarding the broad signal at 2.5 ppm (integral 3.0) noted in the original spectrum,

we attribute this signal to exchangeable protons (OH groups) interacting with residual moisture. In the newly acquired spectrum, the sample was subjected to more rigorous drying prior to analysis. Consequently, this broad signal is no longer observed, confirming that it originated from moisture-dependent exchangeable protons rather than a structural motif of compound 2. The revised spectrum has been included in the revised Figure S1 in the Supporting Information and also shown below.

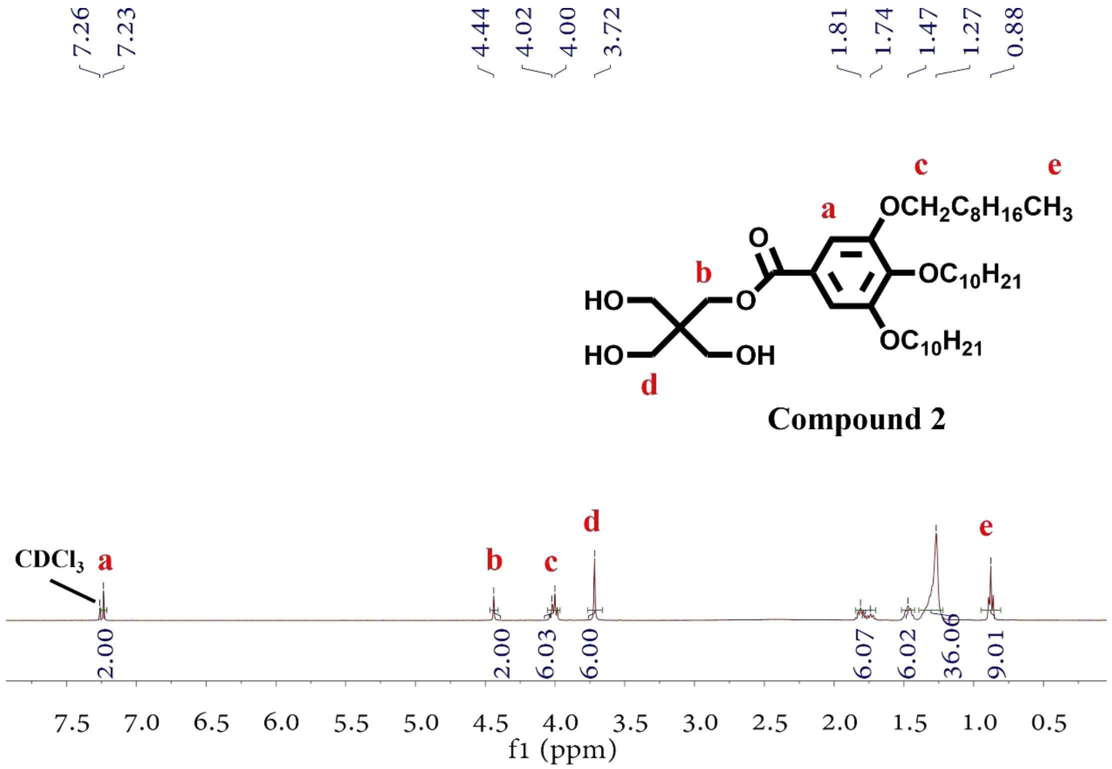

Figure R1. ^{1}H NMR spectra of **Compound 2** (400 MHz in $CDCl_3$).

2. *Similar issues are present in the ^{1}H NMR characterization of the asymmetric dendron shown in Figures S4. Although some signal assignments are provided, the same problems with inconsistent and systematically overestimated integrals persist. The reported positive deviations are significantly larger than expected for properly acquired quantitative ^{1}H NMR spectra.*

Response:

We have also re-acquired the ^{1}H NMR spectrum of the asymmetric dendron (AD) under the same quantitative conditions described above. The revised spectrum includes updated signal assignments and integrations, which are now fully consistent with the expected proton counts within normal experimental uncertainty. The updated data have been provided in the Figure S4 of the Supporting Information and shown below.

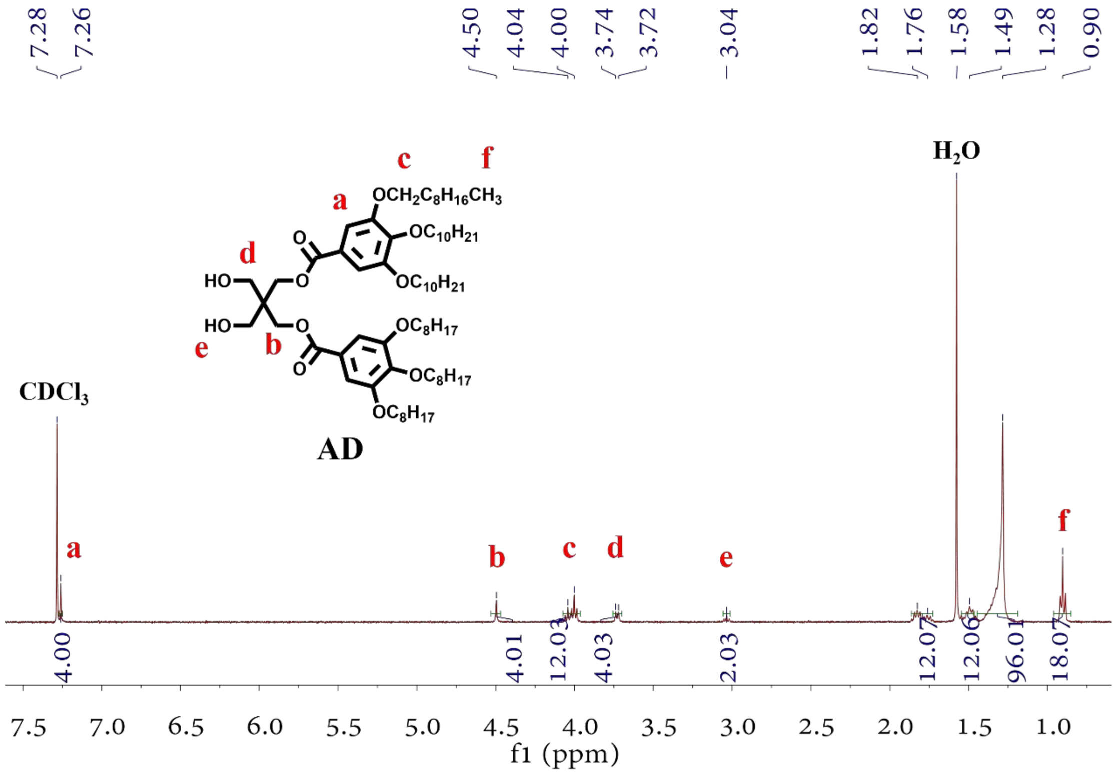

Figure R2. ^{1}H NMR spectra of **AD** (400 MHz in $CDCl_3$). *Reviewer: 2*

Comments:

1. *The manuscript focuses on one degree of asymmetry (C10/C14). Have more asymmetric combinations (e.g., C8/C16 or other extreme mismatches) been tested? Such results would clarify whether: deformability increases monotonically with asymmetry, an upper limit exists beyond which FK stability is lost, or excessive asymmetry produces new phases. Addressing this point would significantly strengthen the mechanistic framework.*

Response:

We also investigated the phase behavior of the other asymmetric dendron, 3-hydroxy2-(hydroxymethyl)-2-(((3,4,5-tris(hexadecyloxy)benzoyl)oxy)methyl)propyl 3,4,5tris(octyloxy)benzoate (C_8C_{16}), which has more significant chain-length asymmetry than AD. As shown in Figure R3a, the in situ variable-temperature XRD data indicate that at this level of asymmetry, the system is unable to form an FK phase. Although liquid-like packing (LLP) was observed during the second heating cycle, only a typical bcc structure was detected upon further heating. This phenomenon where elongation of the hydrophobic chain prevents self-assembly into an FK phase has also been reported in block copolymer systems.[1] The increase in chain length

allows the chains to more effectively fill the intermicellar voids without requiring symmetry breaking or significant chain deformation, thereby favoring the formation of the conventional bcc phase.

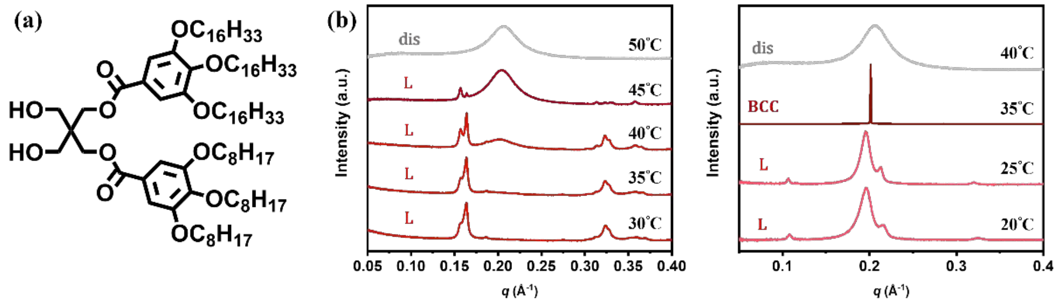

Figure R3. (a) Chemical structure of C_8C_{16}. (b) Temperature-dependent SAXS profiles of C_8C_{16} collected during the first heating and second heating scans.

Reference:

(1) Lewis III, R. M.; Arora, A.; Beech, H. K.; Lee, B.; Lindsay, A. P.; Lodge, T. P.; Dorfman, K. D.; Bates, F. S. Role of chain length in the formation of Frank-Kasper phases in diblock copolymers. *Phys. Rev. Lett.* **2018**, *121*, 208002.

2. *In Figure 4, the (410) reflection intensifies dramatically after two weeks, signaling substantial motif reorientation or deformation accumulation. Yet the simulations in Figure S9 reproduce only the one-week evolution. The reviewer requests clarification:*

What additional structural reorganization occurs between week 1 and week 2 that is absent from the current model? A more explicit discussion would improve the alignment between experiment and simulation.

Response:

We thank the reviewer for the insightful suggestion. To clarify this issue, microbeam SAXS pattern of the AD sample aged for two weeks was also collected in **Figure R4**. When compared the as-prepared AD sample with aged one, it is found that the reorientation of the deformed micelles caused also the re-orientation of the σ-phase lattice on the substrate over time. As shown in the **Figure R4**, after two weeks, the zone

axis of the microbeam SAXS pattern changed from the $[\bar{1}10]$ zone to the $[1\bar{4}0]$ zone, where the (4kl) reflections can be clearly observed. The result indicates that the micellar reorientation

process eventually caused the rotation of the unit cell on the substrate. The rotation is along the c* axis and it pointed the normal direction of the (410) planes toward the beam direction, which resulted in the pronounced enhancement of the (410) reflection of the two-week-aged sample in shown **Figure 3a**.

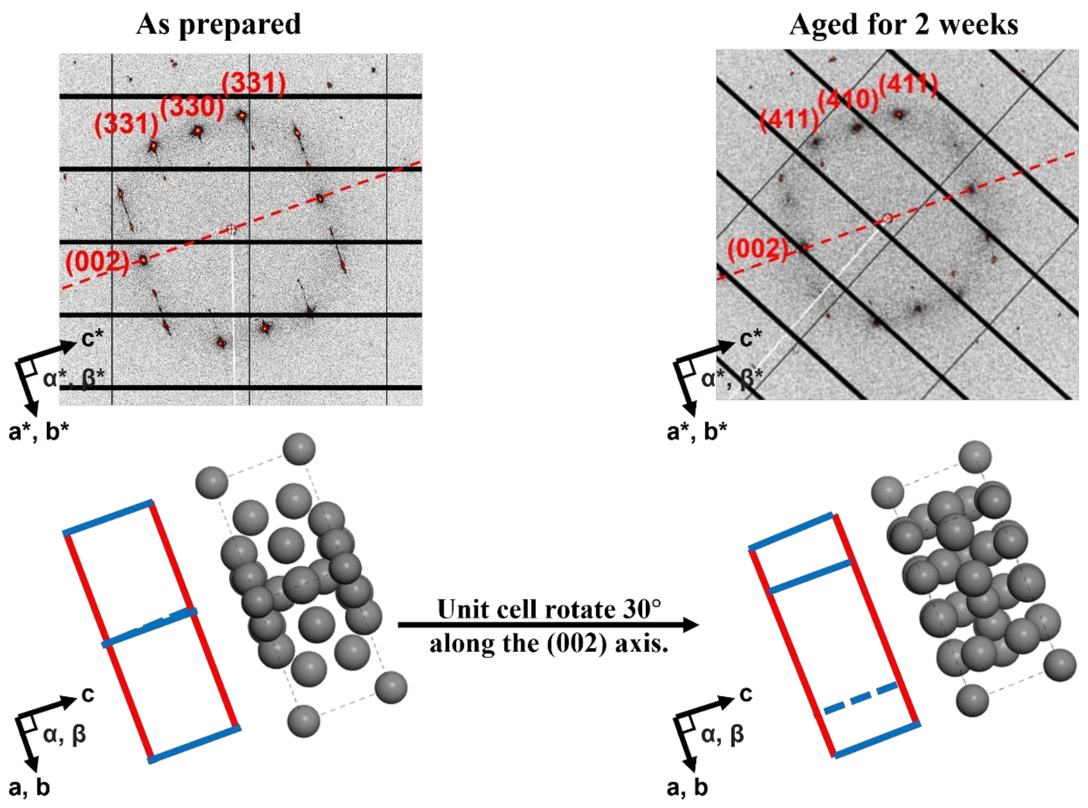

Figure R4. Time evolution of microbeam SAXS patterns of AD σ-phase assemblies, showing that after two weeks the lattice rotates by 30° to align the incident beam along the (410) planes.

***3.** About the long-term stability of BCC at high C12 content, for mixtures with very high C12 content (1:7), is the resulting BCC phase thermodynamically stable? Does it persist upon long-term aging, or does it transform into σ or lamellar phases analogous to those observed in neat AD? Clarification would improve understanding of the phase diagram.*

Response:

We thank the reviewer for suggesting this important thermodynamic experiment. To study the thermodynamic stability of the BCC phase, AD and SD samples were mixed with dodecane in DCM and slowly dried. The resulting mixtures were aged for three weeks before acquiring

SAXS patterns. This procedure was designed to determine whether the mixed samples would, like the pure samples, relax back to the thermodynamically stable lamellar phases after three weeks. The results show that at low dodecane content, such relaxation indeed occurs, as shown in **Figure R5a** and **5b**. In contrast, at high dodecane content, a clear difference is observed: the AD/dodecane mixture remains in the bcc phase and does not revert to the lamellar phase (**Figure R5c**), whereas the SD/dodecane mixture still relaxes back to the lamellar phase (**Figure R5d**).

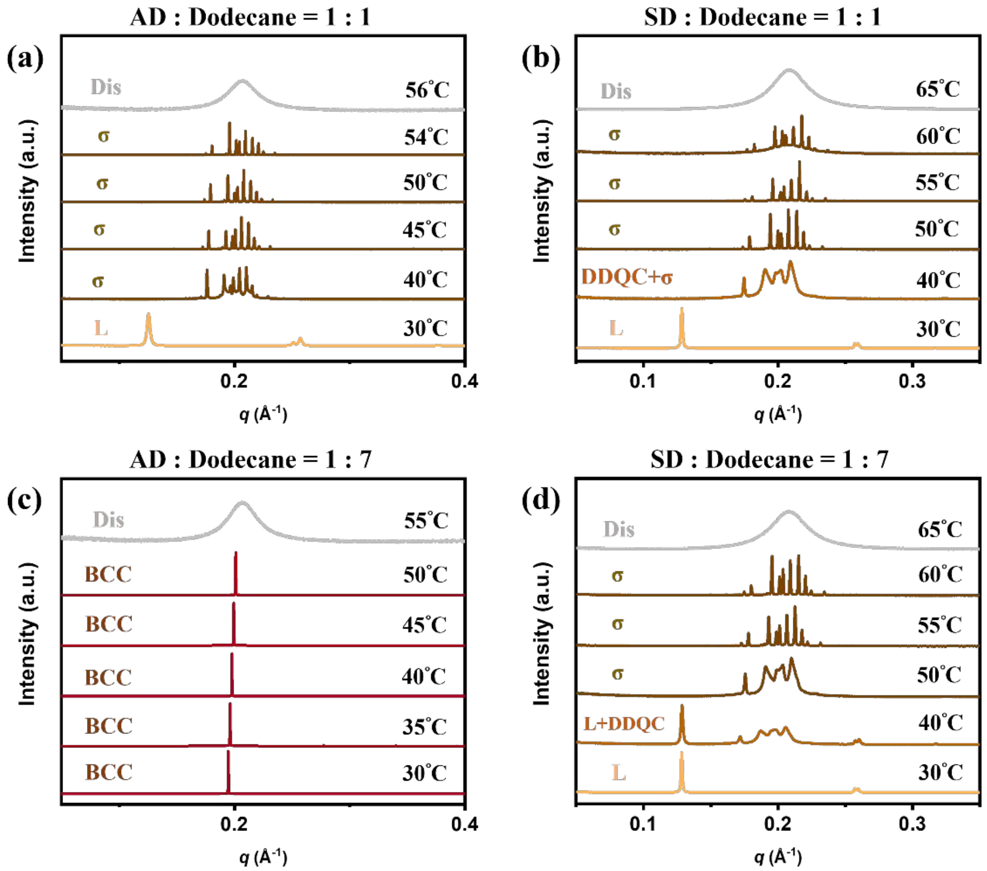

Figure R5. Temperature-dependent SAXS profiles of (a) AD:C_{12} mixtures and (b) SD:C_{12} mixtures at a 1:1 ratio ; (c) AD:C_{12} mixtures and (d) SD:C_{12} mixtures at a 1:7 ratio. The samples were aged for three weeks before acquiring SAXS patterns

4. Cheng and co-workers (Nat. Mater. 2024, 23, 570–576) recently demonstrated unique soft μ and ϕ phase constructed with mesoatom. In the soft ϕ phase, a low fraction of mesoatoms was found to have aspect ratio of ca. 1.2. Although their system is non-amphiphilic and more rigid, lacking the on/off deformability of the present system, and the XRD structural characterization did not reveal the anisotropy of the mesoatoms. A brief acknowledgment would strengthen the manuscript.

Response:

We sincerely thank the reviewer for the suggestion. We has cited this paper as ref. 42 in our manuscript and revised the following paragraph in the introduction of our manuscript.

"Such geometric anisotropy reduces packing frustration and offers an alternative pathway to FK phase stabilization. An unique soft FK phases constructed with mesoatoms, where a degree of shape anisotropy was also recently observed by Cheng and co-workers,[42] showing that equipping the FK phase with structural anisotropy may create possibilities of reaching structural complexity beyond that of current FK phases. However, the mechanism of elongation stabilization has yet to be fully clarified due to the current lack of more sophisticated structural characterization tools and a deeper understanding of the building blocks."

jz-2026-00287u.R2

Name: Peer Review Information for "On and Off Deformability of Supramolecular Micelles in the Soft Frank-Kasper σ Phase."

Second Round of Reviewer Comments

Reviewer: 2

Comments to the Author

The authors have fully addressed the review concerns. This manuscript is now acceptable for publication.

Reviewer: 1

Comments to the Author

The revised version of the paper represents a significant new contribution and should be published as is.

Author's Response to Peer Review Comments:

Thank you for handling our manuscript. We have uploaded a clean version of the publication files without any markups. Additionally, the email addresses of the corresponding authors have been included on the first page of the revised manuscript.
